# Digital Evaluation of Trueness and Fitting Accuracy of Co-Cr Crown Copings Fabricated by Different Manufacturing Technologies

**DOI:** 10.7759/cureus.39819

**Published:** 2023-06-01

**Authors:** Zainab Ali Majeed, Haider Hasan Jasim

**Affiliations:** 1 Department of Conservative Dentistry, College of Dentistry, Ministry of Health, Mustansiriyah University, Baghdad, IRQ; 2 Department of Conservative Dentistry, College of Dentistry, Mustansiriyah University, Baghdad, IRQ

**Keywords:** digital evaluation, internal fit, trueness, milling, 3d printing

## Abstract

Introduction: The dentistry industry has seen a number of exciting new advancements in recent years, many of which have been made possible by the introduction of automated technologies such as computer-aided design and computer-aided manufacturing (CAD/CAM). Despite the fact that these new approaches simplify the fabrication process in favor of decreased material consumption and improved time efficiency, it is possible that they may have an effect on the prosthesis’s fitness, which in turn may affect how long they will last.

Purpose: The purpose of this in vitro study was to evaluate the trueness and fitness of cobalt-chromium (Co-Cr) crown copings fabricated by selective laser melting (SLM), milling, and conventional casting methods.

Materials and Methods: A zirconium die was fabricated and scanned with a laboratory scanner to manufacture the Co-Cr metal copings for three groups (n = 12). In group A, the copings were fabricated by a 3D printing technique called SLM; in group B, the copings were fabricated by the milling technique; and in group C, the copings were fabricated by the conventional lost-wax method. After fabrication, the trueness and the internal fitness of the copings were evaluated using a metrology software program (Geomagic Control X, 3D Systems Inc., Rock Hill, SC). The one-way ANOVA and Tukey’s honestly significant difference test were used to statistically analyze the data.

Results: The highest root mean square (RMS) value of trueness was for CAD/CAM milling, and the highest mean of horizontal gaps was for the casted (lost-wax technique) group. There were highly significant differences in the mean RMS value of trueness and the mean horizontal gaps between the three groups.

Conclusion: The fabrication method of Co-Cr crown copings has an effect on the trueness and fitness of the copings.

## Introduction

Precise fit is a prominent characteristic of the successful service of prosthetic restorations [1-5]. One of the primary factors that affect fitness is trueness, which is the similarity between the design of the prosthesis and the manufactured prosthesis [6-8]. An incorrect intaglio surface of a prosthesis is one of many elements that affect internal and marginal fit; thus, a trueness evaluation is crucial [9,10].

In metal-ceramic restoration manufacturing, lost-wax technique casting [11] is adopted as the golden standard, but although well established, it is challenging, time-consuming, and involves excessive manual steps including tooth preparation, impression, cast production, waxing, investing, and casting [12,13]. It is, therefore, being rapidly replaced by computerized techniques in order to provide automation for the production cycle, ensure minimization of error, and achieve standardized repeated accuracy in the fabrication of prosthetic restorations [14,15]. Such computerized techniques are divided into subtractive and additive [3,16-19]. In subtractive manufacturing, the process involves the subtraction of metal copings from metal solid blocks by cutting with the aid of a computer numerically controlled system. However, residual material cannot be reused, and cutting results in excessive stress on the milling equipment and the end mill [17,18].

In additive manufacturing (3D printing) of metal, the most commonly used method is powder bed fusion [20,21]. There are several different names for this process, including selective laser sintering (SLS), selective laser melting (SLM), direct metal laser sintering (DMLS), and electron beam melting (EBM) [22,23]. The powder bed fusion method adheres to the fundamental principle of producing the product layer by layer and then fusing them together. The chosen cross-sectional area of a powder base material is heated by a heat source that directs its heat there. A source like a powerful laser is focused on a metal powder in accordance with the design of an object. The laser beam then fuses the powder particles one at a time until the 3D object is formed [24]. 3D evaluation is used to assess the accuracy of mass-produced products [25,26]. This saves time over visual examination [26]. Due to the advancement of scanners, it is now possible to conduct an accurate analysis [27]. The distance between certain points can be calculated [28,29], and 3D analysis can be achieved [8,30-33].

Therefore, the trueness and fitness of metal copings fabricated by SLM, milling, and conventional casting techniques were evaluated in this in vitro study. The research hypothesis was that there would be no difference in trueness or fitness between groups.

## Materials and methods

**Figure 1:**
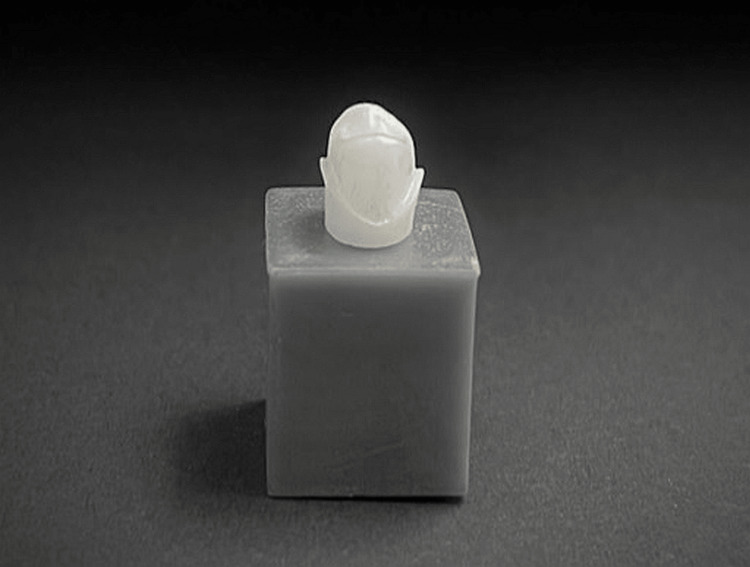
Zirconium die

Zirconium dies with planar occlusal surface reduction, 0.5 mm shoulder finishing line, and 6° convergence angle were manufactured following a complete digital workflow using exocad and Autodesk® 3ds Max® (Figure 1).

The design of the prepared 3D tooth model was sent to the milling machine (model k5, vhf camfacture AG, Ammerbuch, Germany), where one model (maxillary first premolar) was milled from zirconium discs (Zirlux® 16+ white, Henry Schein, Melville, NY).

**Figure 2:**
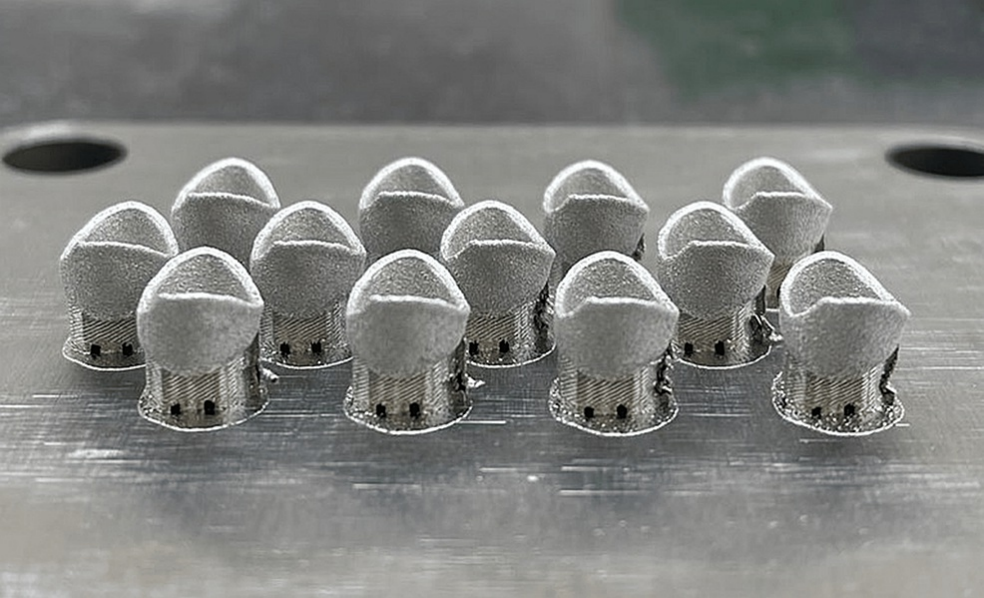
3D-printed copings

A total of 36 (n = 12) cobalt-chromium (Co-Cr) metal copings were manufactured using the 3D printing technique SLM (group A), the milling technique (group B), and the lost-wax method (group C). In the 3D printing technique, the computer-aided design (CAD) data were sent to the 3D printer (ProX DMP 100, 3D Systems Inc., Rock Hill, SC) using SLM. The copings were manufactured, and the laser scanning speed was 7000 mm/s. All the copings were 3D printed from metal powder (LaserForm CoCr (C), 3D Systems Inc., Rock Hill, SC), and the layer thickness was 30 µm (Figure 2).

**Figure 3:**
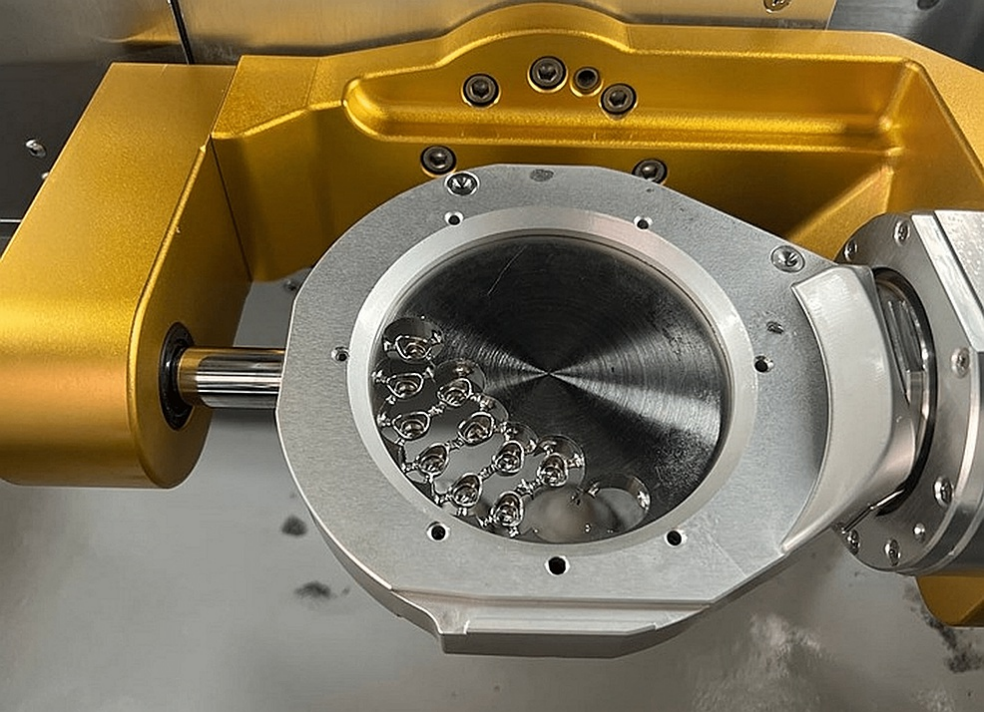
Milled copings

After completing the laser melting process, the annealing was done for 20 minutes in a preheated furnace at 800°C according to the manufacturer’s instructions. After that, the metal copings were detached from their supports and were finished. The CAD data were sent to the milling machine (Arum 5X-500, Doowon, Daejeon, South Korea). Co-Cr metal copings were manufactured by the milling of metal blanks (KERA®-DISC; Eisenbacher Dentalwaren ED GmbH, Rhine-Main, Germany). The CAD data were sent to a 3D printer (Asiga Max, Alexandria, Australia), where 12 samples were 3D printed from castable resin (CURO Cast). The castable resin patterns were sprued, invested, and cast from Co-Cr dental alloy (Kera®C) according to the manufacturer’s instructions (Figure 3).

Trueness analysis

**Figure 4:**
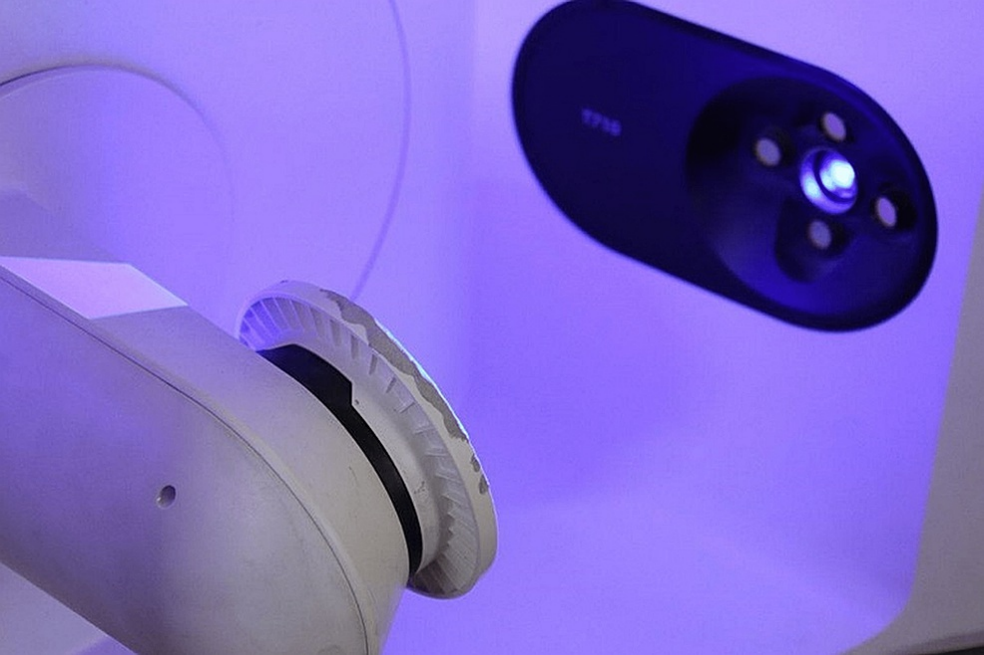
Scan of the inner surface of the copings

Using an optical scanner (Medit T710 desktop scanner, Seoul, South Korea), the inner surface of each coping was scanned. Before each use, the scanner was calibrated in order to ensure precise scanning. Each scan was saved as an STL file for later use in trueness evaluation [34]. A 3D inspection software (Geomagic Control X, 3D Systems Inc., Rock Hill, SC) was used. The coping designed model (CDM) file and the coping scanned model (CSM) file were imported into the software, and the two files were superimposed by initial and best-fit alignment. The 3D compare option was then selected to measure dimensional differences between CDM and CSM, in which tolerance was ± 10 µm and maximum and minimum range was ±100 µm. The data point in the inner region of each scan includes three coordinates (X, Y, and Z). In order to find the trueness value, the distance between each data point in the CDM and its equivalent point in the CSM files was calculated (Figure 4).

Root mean square (RMS) values are used to describe the scanned data’s standard deviation. When the RMS value is small, it means that the overlapping data are in high 3D agreement. Color difference maps are displayed for each 3D comparison when CSM file data are positioned above CDM file data that are shown by the red area in the map, which ranges from 10 to 100 µm. This suggests that the manufacturing did not succeed in reaching the intended position. When CSM file data are positioned below CDM file data, as indicated by the blue zone, which ranges from −10 to −100 µm, it suggests that the manufacturing process went beyond the desired location. The areas that were made perfectly match the green zone, which is less than 10 µm. The color difference map will show more red and blue areas if the RMS value is high, indicating that the 3D agreement of the overlay data is poor. Light body (Express™ Light Body, Fast Set, 3M ESPE) was used to fill the intaglio surface of the copings, and then, the coping was seated on the model and loaded with finger pressure (35). Each coping was seated onto the model under 5 kg (50 N) occlusal force. The coping with the material after its complete polymerization was then removed from the die [35,36].

Using an optical scanner (Medit T710, Seoul, South Korea), the inner surface of each coping with and without the impression material was then scanned. The STL files of the scans were saved, loaded in the software (Geomagic Control X, 3D Systems Inc., Rock Hill, SC), and then superimposed with initial and best-fit alignment. Each superimposed file was sectioned mesiodistally and buccolingually by hypothetical planes, and the fitness was measured along those planes. For each sample, 600 points were selected along the hypothetical planes, and the points were equally spaced in all the samples. A statistical program was utilized for the data analysis using IBM SPSS Statistics, version 26.0 (IBM Corp., Armonk, NY). One-way ANOVA and Tukey’s honestly significant difference test were used for significant difference evaluation between the groups.

## Results

The mean RMS value for trueness was the highest for CAD/computer-aided manufacturing (CAM) milling (44.4 μm), followed by the 3D printed (SLM) group that had 27.2 μm, and the lowest RMS value was for the casted (lost-wax technique) group, which was 18.0 μm (Table 1).

**Table 1:** Mean RMS value (μm) for trueness of the three groups SLM, selective laser melting; CAD, computer-aided design; CAM, computer-aided manufacturing; RMS, root mean square

Groups	N	Mean	Standard deviation	Minimum	Maximum
3D printed (SLM)	12	27.2	2.73	23.0	32.1
CAD/CAM milling	12	44.4	3.66	36.7	50.2
Casted (lost-wax technique)	12	18.0	4.04	11.9	26.8

For the internal fitness, the casted (lost-wax technique) group showed the highest mean internal gap (42.8 μm), followed by the 3D printed (SLM) group that had a mean internal gap of 39.6 μm, and CAD/CAM milling showed the lowest mean internal gap of 33.1 μm. One-way ANOVA test presented very high significant differences at P = 0.000 for the mean RMS value of trueness and mean internal gaps (Table 2).

**Table 2:** Mean internal gaps (μm) of the three groups SLM, selective laser melting; CAD, computer-aided design; CAM, computer-aided manufacturing

Groups	N	Mean	Standard deviation	Minimum	Maximum
3D printed (SLM)	12	39.6	6.13	31.90	54.00
CAD/CAM milling	12	33.1	3.23	27.75	39.40
Casted (lost-wax technique)	12	42.8	6.33	33.55	53.95

The Tukey test was performed for multiple comparisons between different pair of groups to examine the source of difference. The Tukey test for mean RMS values of trueness revealed a significant difference between different groups (P = 0.000). As for the internal gaps, there was a significant difference between CAD/CAM milling and casted (lost-wax technique) group (P = 0.000) and CAD/CAM milling and 3D printed (SLM) (P = 0.01), and there was no significant difference between casted (lost-wax technique) and 3D printed (SLM) groups (P = 0.328).

## Discussion

The null hypothesis was rejected because there was a significant difference in trueness (P = 0.000) and fitness (P = 0.000) between different manufacturing techniques of Co-Cr metal copings. In this study, the results showed that group B (CAD/CAM milling) reported the smallest internal gap between the groups, and these results are in agreement with studies made by various authors [37,38] and in disagreement with other studies [16,39-41]. 

Although group B (CAD/CAM milling) had the smallest internal gap, it had a significantly greater RMS value than the other groups. Thus, the lowest trueness, which means copings manufactured by CAD/CAM milling, had the greatest deviation from the design, so it is reasonable to believe greater RMS value. Thus, lower trueness is attributed to the size and shape of the milling burs, quality of CAD-data CAM’s gathering, and processing capabilities; round edges are formed as a result of the limited resolution of the CAD-CAM imaging system as well as the difficulty in scanning the acute angles while the image is being taken by the scanner and reconstructed by the software program. Light reflects more intensely during the reading process than in flat areas [41-45].

In this study, group C (casted 3D-printed pattern) had the highest internal gap among the three groups, and the results are in agreement with various studies [3,46]. However, it had the lowest RMS value and high trueness value, which means that casted 3D-printed patterns were the closest to the design. This might be attributed to the dimensional accuracy control because if the dental casting investment’s thermal expansion is known, then it is possible to scale the CAD model to account for shrinkage of the casting and create 3D-printed patterns in an optimal size [47,48]. The increase in the internal gap within group A (SLM) may be due to errors that occur during the construction process, segmentation, and also printing [49-51]. The RMS value for SLM copings was low, which suggests good 3D agreement between the design and the SLM-manufactured coping.

In this study, milled copings and casted copings had statically significant differences at P = 0.000, and these results are in disagreement with [16], who measured the internal gap at the marginal, cervical, axial area, and incisal edge of copings made using the DMLS and computer-aided milling (CAM) systems in contrast to casting procedures and discovered that the gap varied greatly with manufacturing methods. The smallest gap was obtained by casting in each of the four measured places. This finding is likely because of the use of different evaluation methods of internal fit, different numbers of points to evaluate the internal fit, and different casting techniques.

The results are also in disagreement with various studies [39,40], which found that copings manufactured by the lost-wax technique had the best fitness. This disagreement may be due to the use of milled castable resin patterns by Abdelhafiz et al. [39], different die shapes, different abutments, and overall dissimilar methodologies used by the researchers. Another finding is the statically significant difference at P = 0.007 between the milled and SLM copings; the SLM showed a higher internal gap than the milled group. This may be because, during fabrication, the contained loose powder is melted by the ambient heat, leading to a poor fit in tiny and intricate structures [52], or because of the rapid melting and cooling of the metal during the SLM process that will eventually affect the dimensions of the metal coping. This is in agreement with [38] and is in disagreement with [53].

There was no statically significant difference (P = 0.157) between the casted and SLM copings; however, the casted group had a slightly higher internal gap that can be attributed to the alloy powder utilized in the SLM method, which has a different composition than the casting material. The alloy for SLM has a lower molybdenum percentage than the alloy that is used for casting. Additionally, the SLM method produces better outcomes while fully eliminating the casting processes and associated human faults [54]. These results are in agreement with other studies [55] and in disagreement with various other results [50,56,57]. For all of the groups, the results of both trueness and fitness were within the normal acceptable range.

## Conclusions

Considering the limitation of this study, the study concluded that CAD/CAM milling group had the smallest internal gap among the three groups; however, it had the lowest trueness, which means that copings manufactured by CAD/CAM milling had the greatest deviation from the design. Casted copings from 3D-printed patterns had the highest internal gap among the three groups; however, it had the lowest RMS value and high trueness value, which means that casted 3D-printed patterns were the closest to the design. The SLM group had an increase in the internal gap compared to CAD/CAM milling; however, it had a low RMS value, which suggests good 3D agreement between the design and the SLM-manufactured coping. All internal gap distances and RMS values for trueness obtained from this study for all of the techniques used to fabricate Cr-Co metal copings are within the acceptable range for clinical use.
